# Allogeneic Hematopoietic Stem Cell Transplantation in Cutaneous T-Cell Lymphomas

**DOI:** 10.3390/cancers12102856

**Published:** 2020-10-03

**Authors:** Maëlle Dumont, Régis Peffault de Latour, Caroline Ram-Wolff, Martine Bagot, Adèle de Masson

**Affiliations:** 1 Department of Dermatology, APHP, Saint-Louis Hospital, F-75010 Paris, France; maelledumont@gmail.com (M.D.); caroline.ram-wolff@aphp.fr (C.R.-W.); adele.demasson@aphp.fr (A.d.M.); 2 INSERM U976, Human Immunology, Pathophysiology and Immunotherapy, Institut de Recherche Saint-Louis, F-75010 Paris, France; 3 Department of Medicine, Université de Paris, F-75010 Paris, France; regis.peffaultdelatour@aphp.fr; 4 Hematology-Bone Marrow Transplantation, APHP, Saint-Louis Hospital, F-75010 Paris, France

**Keywords:** cutaneous T-cell lymphomas, mycosis fungoides, Sézary syndrome, allogeneic hematopoietic stem cell transplantation, lymphomas, review

## Abstract

**Simple Summary:**

Advanced-stage cutaneous T-cell lymphomas are aggressive diseases with frequent disease relapses and a reduced overall survival. Most treatment regimens fail to induce long-term remissions. Allogeneic hematopoietic stem cell transplantation has been associated with treatment-free long-term remissions and holds a potential for cure in this disease but is associated with frequent complications, mostly linked to the development of graft-versus-host disease and infections. Herein, we review the current evidence supporting the use of allogeneic stem cell transplantation in advanced-stage cutaneous T-cell lymphomas.

**Abstract:**

Cutaneous T-cell lymphomas (CTCLs) are non-Hodgkin lymphomas that develop primarily in the skin. They account for almost 80% of primary cutaneous lymphomas. Epidermotropic CTCLs (mycosis fungoides (MF) and Sézary syndrome (SS)) are the most common form of CTCL. The course of the disease ranges from an indolent clinical behavior in early-stage disease to an aggressive evolution in the advanced stages. Advanced-stage disease is defined by the presence of tumors, erythroderma, or significant blood, nodal or visceral involvement. Advanced-stage disease is characterized by frequent disease relapses, refractory disease, a severely impaired quality of life and reduced overall survival. In the last twenty-five years, allogeneic hematopoietic stem cell transplantation (HSCT) has led to prolonged remissions in advanced CTCL, presumably linked to a graft-versus-lymphoma effect and is thus emerging as a potential cure of the disease. However, the high post-transplant relapse rate and severe morbidity and mortality associated with graft-versus-host disease and infections are important issues. Allogeneic HSCT is thus mostly considered in young patients with no comorbidities and an aggressive, advanced-stage CTCL. Allogeneic HSCT gives the best results in patients with a pre-transplant complete remission of the lymphoma. For this reason, one of the challenges is to define the best time to consider allogeneic HSCT in the disease course. Early identification of patients at high risk for progression is important to identify candidates who may benefit from allogeneic HSCT before their disease becomes treatment-refractory. This review describes the role of allogeneic HSCT in CTCL, summarizes the published data and future perspectives in this area.

## 1. Introduction

**Figure 1:**
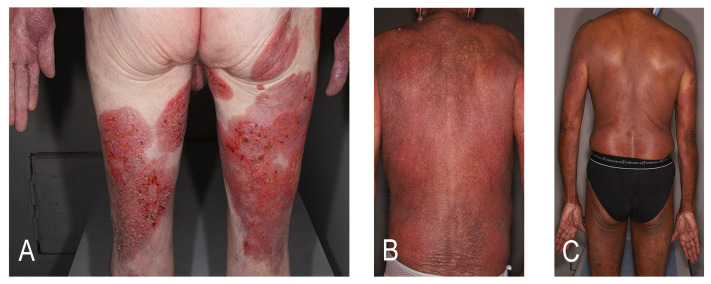
Clinical presentations of advanced-stage disease. (**A**) Mycosis fungoides with tumors (stage IIB); (**B**) mycosis fungoides with erythroderma (stage III); (**C**) Sézary syndrome with significant blood involvement (stage IV).

Cutaneous T-cell lymphomas (CTCLs) are a heterogeneous group of lymphoproliferative disorders defined by the European Organization for Research and Treatment of Cancer (EORTC)-World Health Organization (WHO) classification [1]. CTCLs are characterized by the specific tropism of malignant T-cells to the skin. The various CTCL subtypes are defined by their clinico-pathological characteristics and differ in terms of prognosis. The most common CTCLs, mycosis fungoides (MF) and Sézary syndrome (SS), are epidermotropic lymphomas. MF accounts for around 55% of cases with a 5-year overall survival (OS) rate of 80% whereas SS is a rare leukemic variant accounting for less than 5% of cases with a 5-year OS of 11% [2]. The prognosis is strongly dependent on the staging. More than 80% of early-stage patients with MF will have an indolent course with patches or plaques without extracutaneous involvement [3]. However, almost a quarter of early-stage patients with MF will develop disease progression [4]. Advanced-stage disease is defined by the presence of tumors (stage IIB, Figure 1A), erythroderma (stage III, Figure 1B), or significant blood (Figure 1C), nodal or visceral involvement (stage IV) [3]. Independent prognostic markers have been identified for patients with advanced MF/SS: stage IV, age > 60 years, large-cell transformation (LCT) and increased lactate dehydrogenase [5]. The estimated 5-year survival rate is approximately 40% for advanced-stage disease. The therapeutic choices are thus guided by a stage-based approach. Skin-directed therapies are most often sufficient for early stages, but advanced-stage disease is challenging to treat. Most current therapies have response rates below 50% in advanced-stage disease, with frequent relapses leading to multiple successive treatment lines [6]. According to the EORTC consensus recommendations, allogeneic hematopoietic stem cell transplantation (allo-HSCT) is the only curative option in MF/SS (with the exception of localized radiotherapy for unilesional MF) [7]. The recommendations presented here are mostly based on retrospective studies with a lack of randomized controlled data. The results of a recent meta-analysis might represent the best evidence supporting the use of allo-HSCT as an effective treatment for CTCL [8]. Prolonged remissions with allo-HSCT suggest a graft-versus-lymphoma (GVL) effect [9,10,11,12]. On the other hand, HSCT is associated with a non-negligible high morbidity and treatment-related mortality rate. Graft-versus-host-disease (GVHD) and infections contribute to a nonrelapse mortality of around 20% [8]. Over recent decades, different therapeutic strategies have been studied to promote the GVL effect without increasing the risk of GVHD in order to increase survival and reduce relapse while maintaining a good quality of life. New transplantation protocols, in particular conditioning protocols, are currently being developed with encouraging results, and new sensitive methods to monitor the molecular residual disease before and after allo-HSCT, such as high throughput sequencing of the T-cell receptor beta gene, are now used in CTCL. This review describes the selection of patients, the different types of donors, the pre- and post-transplant therapeutic management, focusing on the published data and future perspectives in the field.

## 2. Selection of Patients for Allo-HSCT 

A large treatment heterogeneity in advanced MF/SS and differences between USA and non-USA centers have been highlighted in a large multicenter retrospective study [13]. With regards to allo-HSCT for CTCL, international recommendations converge. The American guidelines of the National Comprehensive Cancer Network (NCCN) recommend that allo-HSCT may be considered for patients with stage IIB-IV disease that is progressive or refractory to primary treatment options [14]. European guidelines recommend allo-HSCT for young and fit patients with advanced stages of the disease, a low tumor burden at the time of transplantation, and at the same time, a high predictable risk of progression and poor prognosis [7]. These recommendations show that the selection of patients is difficult and is based on several criteria: age and general condition of patients, disease status, and number of prior treatments. Defining the right timing for allo-HSCT in the course of the disease is currently challenging.

In the meta-analysis by Iqbal et al., the median age of allo-HSCT recipients with CTCL ranged from 46.5 to 51.5 years [8]. In a large multicenter retrospective French study, an age under 50 years of the recipient was associated with increased progression-free survival (PFS) (hazard ratio, HR 0.4; *P* = 0.03) and tended to be associated with increased OS (HR 0.3; *P* = 0.05) [15]. However, in a recent American study, patients who were aged ≥65 years at the time of allo-HSCT had similar clinical outcomes compared with younger patients [16]. 

Chronological age must be considered but physiological age might be even more important. A poor performance score (Karnofsky <70) tended to increase nonrelapse mortality (NRM) [17]. 

However, comorbidities have been poorly described in studies on allo-HSCT in CTCLs. In the phase II American clinical trial, patients with pulmonary diffusion capacity <40%, cardiac ejection fraction <30%, history of HIV infection, and Karnofsky performance status <70% were excluded [16]. The hematopoietic cell transplantation comorbidity index (HCT-CI) is an available tool for risk assessment before allo-HSCT [18]. It would be interesting to study this index in patients with CTCL. 

Patient selection is also particularly based on the disease course. In a retrospective analysis of the European group for Blood and Marrow Transplantation (EBMT), advanced-phase disease at allo-HSCT (defined, in this study, as having received four or more prior lines of systemic therapy) was the most important factor for PFS (Relative risk 4.4; 95% confidence interval (CI), 1.7 to 11.4, *P* = 0.002) [11]. This suggests that allo-HSCT should be performed before the disease becomes treatment-refractory. Transformed MF is usually associated with an aggressive disease course and short durations of response to therapies [19], and for this reason, allo-HSCT should probably be proposed early in the course of transformed MF. In a French retrospective study on allo-HSCT in advanced CTCL, including 54% of patients with transformed MF, the estimated 2-year overall survival was 57% (95% CI: 0.41–0.77) suggesting that allo-HSCT is a valuable treatment option in advanced-stage CTCL including transformed MF [15]. 

In advanced CTCL, disease status and tumor burden need to be closely monitored. If possible, transplantation should be performed in pre-transplant complete remission or near-complete remission. Patients in complete response (CR) or very good partial response (VGPR) had a lower cumulative incidence of relapse compared with those with a higher disease burden (*P* = 0.04) [11]. A weak residual tumor burden before transplantation is associated with increased PFS (HR 0.3; 95% CI, 0.1 to 0.8; *P* = 0.01) [15]. High-throughput T-cell receptor sequencing of the TCRB gene allowed the assessment of the minimal residual disease (MRD) with a good sensitivity [20] and identified aggressive early-stage MF [21]. High-throughput sequencing (HTS) could thus help identify candidates who may benefit from allo-HSCT before their disease becomes treatment-refractory. Indeed, the purpose of monitoring is to help define the right timing for transplantation. A review of the early studies on allo-HSCT in CTCL showed that results could be further improved if the procedure was performed earlier in the disease course, before patients were very heavily treated [22]. Ten years later, in a retrospective study on patients with a median time from diagnosis to allo-HSCT of 46 months (range 9-309), authors compared OS in patients with time from diagnosis to transplant below 46 months versus above 46 months. The patient group with time from diagnosis to transplant < 46 months had a longer OS at 1 year (*P* < 0.04) [12]. These results, although derived from retrospective studies, suggest that allo-HSCT should be considered early in the course of the disease.

## 3. Bridging Therapies to Allo-HSCT

With a median number of four prior treatments before allo-HSCT [8], bridging therapy is a key step in order to achieve complete remission before transplantation. 

Total-skin electron beam radiation therapy (TSEBT) debulks the skin just before transplantation [23]. In a prospective study of 47 patients, Hosing et al. treated 42 patients with more than 10% of body surface area involved with a median dose of 32 cGy (range, 12-36) within 8 to 9 weeks. Radiation was completed 2 to 3 weeks before the transplant, allowing healing of the skin at the time of transplant [24]. As we have seen previously, tumor burden is correlated with prognosis [17]. Low dose radiotherapy can eradicate the malignant T-cell clone in MF patches and plaques and might thus reduce the risk of post-transplant relapse in skin [25]. 

Over the past few years, brentuximab vedotin (BV) has appeared as a good candidate as a bridge to allo-HSCT. BV is an antibody–drug conjugate composed of a humanized anti-CD30 monoclonal antibody and the antimicrotubule and cytotoxic agent monomethyl auristatin E. It has been associated with significant improvement in objective response lasting at least 4 months compared to the physician’s choice of methotrexate or bexarotene in CD30-positive MF or primary cutaneous anaplastic large-cell lymphoma in a multicenter, prospective, randomized phase III study [26]. Of note, patients with SS were not enrolled in this phase 3 study. Schneeweiss et al. described the case of a woman with transformed folliculotropic MF who became eligible for transplantation after four cycles of BV [27]. In a retrospective series of T-cell non-Hodgkin lymphomas including five patients with transformed MF, 4 of these 5 patients treated with a standard dose of BV of 1.8 mg/kg every 3 weeks were in partial response (PR) before allo-HSCT. For the entire cohort, the median time between the end of bridging treatment and allo-HSCT was 44 days (7-206). The main toxicity of BV was peripheral neuropathy (mostly sensory) and occurred in the vast majority of the patients after four cycles [28]). In CTCL patients, the median OS rate was 80% but with a short median follow-up of 13.5 months (range 1.1–39.8) [29]. Interesting results have been recently reported on the effectiveness of BV before but also after allo-HSCT in the management of transformed MF [30]. In a French retrospective cohort of nine patients, BV plus bendamustine allowed subsequent allo-HSCT in two patients with transformed SS [31]. Thus, BV may be a good option, alone or in combination, as a bridge therapy for allo-HSCT in CTCL with poor prognosis. BV has also shown efficacy in patients with CD30-negative disease [32]), probably due to the variability in CD30 expression from one lesion to another in the same patient [33].

Pre-transplant treatments should allow complete or partial remission with no significant toxicity but also without negatively influencing the post-transplant evolution. For example, mogamulizumab is a humanized anti-CCR4 monoclonal antibody that has shown to increase PFS compared to vorinostat in advanced CTCL in a phase 3 randomized controlled study [34]. Long-term remissions and auto-immune side-effects have been associated with the use of mogamulizumab [35], probably due to the depletion of mature regulatory T-cells. Indeed, CCR4 is expressed on malignant T-cells but also on normal mature regulatory T-cells. Regulatory T-cells were depleted for several months after the administration of mogamulizumab and the depletion of regulatory T-cells was associated with a higher risk of GVHD [36]. In adult T-cell leukemia/lymphoma patients, a short interval between mogamulizumab and allo-HSCT doubled the risk of acute GVHD [37]. Dai et al. described a potential association of mogamulizumab and GVHD in MF and SS but further studies are needed to confirm these findings [38].

Another strategy is based on a sequential approach with autologous HSCT shortly followed by allo-HSCT. Autologous HSCT requires high doses of chemotherapy and/or radiotherapy to minimize the tumor burden and then allow the reinfusion of autologous hematopoietic stem cells. The majority of the cases achieved a CR but early relapse often followed. One of the risks of autologous HSCT is the reinfusion of tumor cells that could contaminate the graft. In a meta-analysis performed to compare the outcome of allogeneic versus autologous HSCT in patients with MF/SS, allo-HSCT offered a longer overall and disease-free survival, likely because of a GVL effect [39]. Thus, some authors have been interested in a sequential approach with auto then allo-HSCT in patients with a high tumor burden. Gabriel et al. showed a GVL effect after early relapse following reduced-intensity sibling allo-HSCT for a relapsed cytotoxic variant of MF treated 6 months earlier with an autograft [40]. Plachouri et al. reported a complete durable remission of a fulminant cutaneous aggressive epidermotropic CD8^+^ cytotoxic T-cell lymphoma after autologous and allo-HSCT. Because of multiple skin ulcerations and a high risk for severe infectious complications, allo-HSCT was not considered as the first salvage option [41]. Autologous HSCT has been abandoned and is not anymore recommended in the international guidelines [7]. A sequential approach could nevertheless be considered as a therapeutic option for specific rare cases with a high tumor burden.

## 4. Different Conditioning Approaches and Types of Donors

In 2010, Duarte et al. identified the conditioning regimen and donor type as drivers of the outcome of allo-HSCT in MF/SS [11]. Two types of conditioning are commonly used in allo-HSCT. A myeloablative conditioning regimen often combines total body irradiation with alkylating agents to induce aplasia. In order to reduce prolonged cytopenias, immunoablative conditioning called reduced-intensity conditioning (RIC) regimens have been developed. Initially, RIC regimens were mostly used in CTCL in older patients and those with comorbidities [22]. The meta-analysis of Iqbal et al. highlighted that RIC regimens were now commonly prescribed (76%) despite the relatively young age of patients in most studies (range, 46.5–51.5 years) [8]. Indeed, large studies about allo-HSCT in CTCL showed good results with RIC. In the European multicenter study, 60 patients were treated with RIC (*n* = 44) or with a myeloablative regimen (*n* = 16). The RIC regimen decreased NRM (RR 4.7, *P* = 0.008) without being associated with higher relapse/progression (REL) [11,17]. Lechowicz et al. reported a similar NRM and OS between RIC and myeloablative regimens [42]. Today it seems appropriate to treat CTCL patients who undergo allo-HSCT with RIC regimens. 

Donor can be related (sibling) or unrelated, and matched or mismatched. With regards to CTCL, matched related donors were more common in studies [8]. Duarte et al. found that allo-HSCT with matched unrelated donors had a significantly lower PFS and OS compared to sibling donors [11,17]. Weng et al. found no difference in the incidence of GVHD or OS between sibling and unrelated donors [16]. Because fully matched donors may not be available, the use of haploidentical donors is currently being explored, especially since this technique improved significantly in the last 15 years with the introduction of post allo-HSCT cyclophosphamide as GVHD prophylaxis. In a meta-analysis of 30 studies on adults with hematological malignancies, haploidentical stem cell transplantation was associated with increased mortality compared with sibling donors, similar mortality compared with matched unrelated donors and reduced mortality compared with mismatched unrelated donors [43]. Further studies including haploidentical donors for allo-HSCT in CTCL are needed to define the place of haploidentical transplantation when neither sibling nor matched unrelated donors are available. 

Three graft sources are possible in allo-HSCT: mobilized peripheral blood stem cells, bone marrow grafts and cord blood transplantation. Mobilized peripheral blood stem cells are the preferred graft source (78%) [8]. This procedure is based on obtaining G-CSF-stimulated hematopoietic stem cells from peripheral blood via apheresis. The advantages are that more cells are collected but also that these transplants contain more mature T-cells than bone marrow transplants. Bone marrow graft is a more invasive method for the donor as it requires bone marrow aspiration under general anesthesia. Data with cord blood transplantation in CTCL are limited but have been mostly reported in Japanese cases [15,42,44,45].

During conditioning, in vivo T-cell depletion can be used to reduce the incidence of GVHD. Two depleting agents are used: rabbit anti-thymocyte globulin (ATG) or alemtuzumab. Alemtuzumab is a T-cell-depleting monoclonal antibody directed against CD52, an antigen expressed on most malignant B- and T-cells. The effector mechanism includes antibody-dependent cellular cytotoxicity (ADCC). In a phase 2 study, the overall response rate was 55% in patients with advanced MF/SS [46]. Alemtuzumab has the advantage that it can be delivered at very low doses subcutaneously with a good toxicity profile and high response rate [47]. In SS, alemtuzumab induced long-term remissions but was poorly effective in MF and transformed CTCL, and 5 out of 39 patients developed large-cell transformations in skin during alemtuzumab treatment [48]. Indeed, MF is a malignancy of skin resident effector memory T-cells. Therefore, the absence of ADCC effector cells (neutrophils, and natural killer cells) in the skin could prevent alemtuzumab-mediated ADCC of skin tumor cells in alemtuzumab-treated MF patients [49]. Alemtuzumab induces a long-lasting peripheral blood T-cell depletion and could prevent the recruitment of antitumor effector T-cells to the skin and thus the T-cell mediated graft-versus-tumor effect after allo-HSCT in MF. These data suggest negative effects on the outcome of the disease using alemtuzumab in allo-HSCT in MF, although we lack specific literature data. The most commonly reported agent is ATG, the use of which depends on local protocols. Duarte et al. highlighted a higher risk of relapse with T-cell depletion (HR 2.48; *P* = 0.0207) [11,17]. The French study of De Masson et al. confirmed that ATG is associated with a decreased PFS (*P* = 0.04) [15]. However, in a prospective study, authors were not able to show an increased risk of relapse with the use of in vivo T-cell depletion with ATG. Patients received ATG only in unrelated or mismatched grafts [24]. The increased relapse rate with ATG suggests a role for T-cells in the GVL effect in CTCL. Indeed, if the GVL effect is linked to the donors’ T-cells, and if depletion of the donors’ T-cells is induced in the pre-transplant phase, a higher frequency of post-transplant relapse can be expected. Thus, an increase in the incidence of post-transplant relapse in patients with pre-transplant T-cell depletion is in favor of a T-cell-related GVL effect. In vivo T-cell depletion should probably be avoided, at least in patients without a high risk of GVHD. 

New conditioning approaches have recently emerged. A novel nonmyeloablative allogeneic transplantation strategy has been used in a phase II study including 35 patients (13 MF and 22 SS patients) with advanced-stage disease [16]. Patients were treated with a unique conditioning using TSEBT (target dose of 30 to 36 Gy), total lymphoid irradiation and ATG. This regimen provided consolidative disease control in the skin with TSEBT while total lymphoid irradiation and ATG conditioning had low GVHD (the 2-year incidence of moderate/severe chronic GVHD was 32%) and NRM (the 2-year NRM was 14%). This alternative strategy was effective in time with a median post-transplant follow-up of 5.4 years; the 5-year OS rate was 56%. Authors highlighted that the major disadvantage was the slower and sometimes incomplete engraftment. Other approaches are being evaluated in patients with other hematologic malignancies to improve the engraftment and may offer interesting perspectives in allo-HSCT in CTCL, such as the use of a very low dose total body irradiation in addition to total lymphoid irradiation and anti-thymocyte globulin (NCT03734601).

## 5. Evolution Post Allo-HSCT

Stem cell engraftment was good and fast in most studies [11,15,42]. The cumulative incidence of sustained neutrophil engraftment was 94% at a median of 14 days post-transplantation in the European study [11]. Results were similar in the American and the French studies with neutrophil engraftment achieved in 95% of patients at day 28 and in 91% of patients at day 30, respectively [15,42]. Patient deaths have been described prior to engraftment due to infections [24]. During this period, patients should be closely monitored. Blood transfusion support may also be necessary until the platelet engraftment is effective. 

NRM is mostly due to GVHD. GVHD prophylaxis consists of immunosuppressive agents (cyclosporine and mycophenolate mofetil or methotrexate in most cases). The incidence of acute GVHD is quite variable across cohorts. In a prospective study, the most commonly involved organ was the skin [24]. The cumulative incidence of chronic GVHD ranged from 43 to 48% at 2 years in major studies [11,15,42]. Lower incidence rates (28 and 32%) have been reported in the MD Anderson Cancer Center study and the Stanford clinical trial [16,24]. Authors suggested that the use of TSEBT as part of the treatment regimen could maybe explain these results [24]. In the European study, there were no statistically significant differences in the cumulative incidence of acute or chronic GVHD by patient age at allo-HSCT, graft source, type of conditioning, or type of donor [11]. 

Relapse remains the most common complication. About half of the patients relapsed [8] within the first year after transplantation. The low rate of late relapse/progression could be linked to a GVL effect and can be explained by the time needed to reconstitute a lymphocyte repertoire allowing the GVL effect to take place. As previously discussed, the effect of T-cell depletion on the incidence of relapse is also consistent with a T-cell-related GVL effect in CTCL. In 2000, Burt et al. described the first case of second clinical and histological remission following post-transplant relapse and the withdrawal of immunosuppression medication after allo-HSCT for advanced MF, providing evidence for a GVL effect [9]. Since then, the management of relapse has been based on a three-step therapeutic strategy. 

As a first step, the treatment of relapse involves the reduction and rapid discontinuation of immunosuppression (IS) in the absence of GVHD, which contraindicates this attitude. In a prospective study, many patients with relapse responded to immunomodulation and achieved durable remissions [24]. In a recent case report, Hosoi et al. managed a SS patient with hematological relapse within one month after allo-HSCT. Intermittent fever developed without infection after the discontinuation of IS. The chimeric analysis of T-cells showed that complete donor chimera in the T-cell fraction was achieved after IS discontinuation allowing a long-term remission of the disease [50]. 

Then, when necessary, donor lymphocyte infusions (DLIs) may enhance GVL effects, but, on the other hand, increase GVHD. In a multicenter UK study of GVHD in various hematological malignancies, most patients developed GVHD following DLI (71%) but relapse was significantly less frequent in those receiving pre-emptive DLI [51]. In 2008, Cudillo et al. described the case of an SS patient with a disease relapse after RIC allo-HSCT who was successfully treated with DLI confirming that Sézary T-cells can be targeted by donor-derived immune cells [12]. Data of the European study showed that half of the relapsing patients achieved a new CR with DLI [11]. Since then, several studies have reported relapses successfully treated with DLI [15,52]. In a recent American study, responses were variable: of the eight patients who had DLI, 3 achieved CR, 2 achieved PR, 2 had stable disease and 1 had progressive disease [16]. In the French study, most relapses involved only the skin. Some patients achieved remission for 8 or 10 years without treatment despite a post-transplant relapse followed by DLI [15]. In the largest cohort study investigating DLI in patients who relapsed after allo-HSCT for primary CTCL, the objective response rates to DLI were around 62%. The median best response duration to DLI was 181 days and the median time before the relapse after DLI was 405 days. Almost half of the patients presented with GVHD after DLI, of which three cases were chronic GVHD. The authors concluded that DLI appeared to be an effective treatment in cases of patient relapse after allo-HSCT for primary CTCL and should be considered in the management of post-transplant relapse whenever possible [53]. 

Finally, one of the possibilities is the resumption of systemic treatment. In one of the earliest reviews about allo-HSCT in CTCL, the authors insisted on the development of newer drugs that combine a strong prophylactic and therapeutic effect against GVHD with efficacy against CTCL which may help control the severity of GVHD while contributing to disease control [22]. In the French study, 6 of the 19 patients who relapsed had a second prolonged complete remission after post-transplant salvage therapy. Treatments of post-transplant disease relapse were disparate. Patients received DLI alone or combined with interferon or local radiotherapy, carmustine, bortezomib or local radiotherapy only [15]. In the Stanford study, the choice of systemic therapy depended on patient’s disease status and previous response to a specific agent(s) [16]. 

Based on a successful case report, some authors have suggested that the combination of DLI and systemic PUVA/bexarotene might be an interesting immunological approach in patients with relapsed CTCL after allo-HSCT. The purpose was to enhance GVL activity with systemic PUVA and bexarotene by inducing the apoptosis of tumor cells which in turn would increase the tumor-antigene stimulation of the transferred donor lymphocytes [54]. No further studies have studied this strategy. The use of PUVA after transplant must be carefully considered, as allo-HSCT recipients are at a high risk of nonmelanoma skin cancer [55]. 

Histone deacetylases (HDACs) are enzymes that interfere with immune surveillance. Vorinostat is a HDAC inhibitor that could prevent GVHD. In a phase 1/2 study, the administration of vorinostat in combination with standard GVHD prophylaxis after related donor RIC HSCT reduced severe GVHD [56]. Shiratori et al. described two patients in relapse with chronic GVHD treated with vorinostat still alive in PR [57]. The authors are conducting a phase I study on post-transplant vorinostat therapy for the prevention of GVHD assuming that the use of vorinostat could control GVHD and prevent relapse of the hematological malignancy at the same time (NCT00810602). 

As previously discussed, BV could be an interesting approach. Well tolerated after transplantation, BV showed effectiveness after allo-HSCT in the management of follicular MF and also transformed MF [30,58]. Larger studies are needed to confirm this effect. 

A close patient follow-up is essential after transplantation in order to adapt treatments (immunosuppressive drugs, infectious prophylaxis and others) as best as possible. Data from skin biopsies are valuable tools. Many patients develop erythematous skin lesions following transplantation and diagnosis of cutaneous GVHD can be a real challenge [59]. Differential diagnoses such as drug reactions and recurrent disease can indeed have a very similar aspect. Menter et al. described the histopathology and immunohistochemistry findings in various biopsies after allo-HSCT in 28 patients with CTCL at Hammersmith Hospital [60]. The authors insisted on three points that could help in diagnosis. First, an immunohistochemistry panel for T-cell antigens is very helpful in making the distinction between GVHD and recurrent lymphoma. The upregulation of HLA-DR in the involved epidermis favors GVHD. Lastly, changes of keratinocytes frequently seen in GVHD are not seen in recurrent lymphoma. In this context, the HTS of T-cell receptors (TCRs) could be an extremely valuable tool to detect recurring disease in skin after allo-HSCT. 

Emerging technology such as the HTS of T-cell receptors (TCRs) has provided a tool to study important aspects of allo-HSCT; in particular, to better understand the GVL effect in CTCL. Weng et al. observed a delayed clearance of the malignant clone in three cases suggesting a slow but persistent GVL effect. Besides, the timing of achieving molecular remission was not entirely associated with full donor T-cell chimerism. Thus, the persistence of malignant clone in the skin several months after achieving molecular remission in the blood in one patient implied a different kinetics of GVL effect in the skin and in the blood [20]. In a recent American cohort, MRD detected by this method was associated with a higher cumulative incidence of progression or relapse [16]. The HTS of TCRs can monitor response to therapy in MF/SS and help determine clinically meaningful molecular remission. 

The use of immunoregulatory drugs before or after allo-HSCT in CTCL may improve the outcome. For example, PD-1 inhibitors have been used in relapsed Hodgkin disease after allo-HSCT, although the risk of GVHD must be carefully weighted [61]. Recently, relapsed and refractory MF and SS were treated with pembrolizumab in a multicenter phase 2 study [62]. Treatment response did not correlate with expression of PD-L1, total mutation burden, or an interferon-γ gene expression signature. Interestingly, the flare reaction was correlated with a high PD1 expression on Sézary cells but did not associate with the subsequent clinical response or lack of response. These data suggest that immune checkpoint inhibitors could be used as a salvage treatment after allo-HSCT in relapsed CTCL, although patients should be carefully monitored for GVHD, as observed with post-allo-HSCT nivolumab used as a salvage therapy in Hodgkin lymphoma [63]. 

## 6. Best Practice Set of Recommendations

Selection of patients for allo-HSCT: 

Patients with advanced-CTCL including transformed MF. 

Identify aggressive early-stage and monitor with HTS of TCR to help measure the molecular residual disease and define the right timing for transplantation.

When and how to condition patients: 

Choose the right treatments to achieve pre-transplantation CR or near-CR: TSEBT or brentuximab-vedotin (for instance). 

Promote RIC (immunoablative) regimen. 

In vivo T-cell depletion should be avoided, at least in patients without a high risk of GVHD.

Donor selection:

Favor sibling donor or, if unavailable, a matched unrelated donor. Given the available data to date, a haploidentical donor should be considered when neither sibling nor matched unrelated donors are available.

Early management after allo-HSCT:

Limit the risk of infections: sterile room; anti-infectious prophylaxis (antibacterial, antiviral and antifungal treatments). 

Reduce the risk of GVHD with IS: cyclosporine and mycophenolate mofetil or methotrexate.

Blood transfusion support may be necessary until the platelet engraftment is effective.

Follow-up: 

Gradual reduction in IS in the year and then discontinuation in the absence of GVHD.

New blood group card corresponding to the donor’s blood group. 

Update the vaccination status.

Relapse management:

Reduction and rapid discontinuation of IS in the absence of GVHD. 

Discuss DLI to enhance GVL effects considering the risk of GVHD. 

Resumption of systemic treatment (Brentuximab vedotin; HDAC).

## 7. Conclusions and Perspectives

New therapeutic approaches are being studied in CTCL such as targeted therapies, combination treatments and immunotherapy, but none to date have a proven curative effect. Allo-HSCT is, today, the only potential curative option in advanced MF/SS. The reported outcomes in a recent meta-analysis were promising [8]. The pooled post-allograft OS rate was 59% (95% CI, 50 to 69%) and the PFS rate was 36% (95% CI, 27 to 45%). On the other hand, the high incidence of post-transplant complications underlines the complexity of allo-HSCT. The pooled relapse rate was 47% (95% CI, 41 to 53%) and the nonrelapse mortality reached 19% (95% CI, 13 to 27%) often within the first year after transplantation (Table 1). The quality of life is also strongly altered with long hospital stays associated with heavy monitoring (very regular blood tests, skin biopsies, repeated imaging such as CT scans), infectious complications, GVHD, systemic therapies, etc. Severe morbidity and mortality should lead to the use of this treatment in patients at a high risk of progression. The early referral of eligible patients to a hematology department is essential to prepare the patient for a potential transplant. A close collaboration between hematologists and dermatologists makes it possible to act promptly and to perform allo-HSCT at the optimal time. 

Novel strategies are being explored to enhance the GVL effect while reducing relapse and nonrelapse mortality by limiting GVHD and avoiding toxicity. Among them, chimeric antigen receptor (CAR) T-cells are autologous T-cells that are genetically modified to express a chimeric antigen receptor (CAR). Patients are injected with these genetically modified T-cells that are activated and directed against tumor cells. These T lymphocytes possess intracytoplasmic-activating motifs (ITAMs, immunoreceptor tyrosine-based activation motifs) so that they are highly activated when they encounter the tumor antigen and induce lysis of the tumor cells. Thus, this type of treatment could significantly reduce the pre-transplant tumor burden in CTCL, which is known to be correlated w the prognosis [15,17]. In addition, there are CAR T-cells expressing suicide genes, allowing their elimination in vivo in order to limit their toxicity. Indeed, serious adverse events (neurological side-effects and cytokine release syndrome) and resistance to CAR T-cells are still important limits. Particularly developed in B-cell lymphomas, data are limited in T-cell lymphomas and there are no published human data in CTCL but this treatment, followed by allo-HSCT to avoid prolonged T-cell depletion, has been proposed. CAR T-cells followed by allo-HSCT may allow for hematological rescue following CAR T-cell mediated disease clearance [64]. 

The role of allo-HSCT in CTCL is becoming better defined but future prospective controlled trials with a high level of evidence are needed. A national hospital clinical research program (PHRC) coordinated by the French Study Group on Cutaneous Lymphomas and the French Society of Bone Marrow Transplantation and Cellular Therapies is currently studying allo-HSCT in advanced CTCL with poor prognostic factors in a prospective controlled study (CUTALLO, NCT02520908). This study will better define the place and optimal timing of allo-HSCT in these diseases. 

## Figures and Tables

**Table 1:** Main studies on patients with cutaneous T-cell lymphomas treated with allogeneic stem cell transplantation.

Ref.	Number of Patients	Median Age (Range)	Pathology	Source of Stem Cells	Conditioning Regimen	Outcomes	Complications	Median Follow-Up
Duarte et al., J Clin Oncol, 2010 and 2014 [11,17]	60	44 (13–63)	MF = 36 SS = 24	PB = 50 BM = 10	MAC = 16 RIC = 44	7 year, OS 44% PFS 30%	7 year, Relapse 45% NRM 22%	7 years
Duvic et al., J Clin Oncol, 2010 [23]	19	50 (21–63)	MF = 5 SS = 14	PB = 14 BM = 5	NMA = 19	2 year, OS 79% PFS 53%	1.7 year, Relapse 37% NRM 21%	1.7 years
De Masson et al., Haematologica, 2014 [15]	37	44 (9–63)	MF = 26 SS = 55 Others = 6	PB = 32 BM = 5 CB = 2	MAC = 12 RIIC = 25	2 year, OS 57% PFS 31%	2 year, Relapse 56% NRM 18%	2.4 years
Lechowicz et al., Bone Marrow Transplant, 2014 [42]	129	48 (22–72)	Not available	PB = 107 BM = 18 CB = 4	MAC = 46 RIC/NMA = 83	5 year, OS 32% PFS 17%	5 year, Relapse 61% 2 year, NRM 22%	3.3 years
Hosing et al., Ann Oncol, 2015 [24]	47	51.5 (19–72)	MF = 34 SS = 13	PB = 35 BM = 12	MAC = 3 NMA = 2 RIC = 42	4 year, OS 51% PFS 26%	2 year, Relapse 50% NRM 17%	2 years

CB: cord blood; BM: bone marrow; MAC: myeloablative conditioning; MF: mycosis fungoides; NMA: nonmyeloablative; NRM: nonrelapse mortality; OS: overall survival; PB: peripheral blood; PFS: progression-free-survival; RIC: reduced intensity conditioning; SS: Sézary syndrome.
